# Targeting Akt in cancer for precision therapy

**DOI:** 10.1186/s13045-021-01137-8

**Published:** 2021-08-21

**Authors:** Hui Hua, Hongying Zhang, Jingzhu Chen, Jiao Wang, Jieya Liu, Yangfu Jiang

**Affiliations:** 1 State Key Laboratory of Biotherapy, Laboratory of Stem Cell Biology, National Clinical Research Center for Geriatrics, West China Hospital, Sichuan University, Chengdu, China; 2 State Key Laboratory of Biotherapy, Laboratory of Oncogene, Cancer Center, West China Hospital, Sichuan University, Chengdu, 610041 China; 3 School of Basic Medicine, Chengdu University of Traditional Chinese Medicine, Chengdu, China

**Keywords:** Akt, Biomarker, Cancer, Cell signaling, Precision therapy, Targeted therapy

## Abstract

Biomarkers-guided precision therapeutics has revolutionized the clinical development and administration of molecular-targeted anticancer agents. Tailored precision cancer therapy exhibits better response rate compared to unselective treatment. Protein kinases have critical roles in cell signaling, metabolism, proliferation, survival and migration. Aberrant activation of protein kinases is critical for tumor growth and progression. Hence, protein kinases are key targets for molecular targeted cancer therapy. The serine/threonine kinase Akt is frequently activated in various types of cancer. Activation of Akt promotes tumor progression and drug resistance. Since the first Akt inhibitor was reported in 2000, many Akt inhibitors have been developed and evaluated in either early or late stage of clinical trials, which take advantage of liquid biopsy and genomic or molecular profiling to realize personalized cancer therapy. Two inhibitors, capivasertib and ipatasertib, are being tested in phase III clinical trials for cancer therapy. Here, we highlight recent progress of Akt signaling pathway, review the up-to-date data from clinical studies of Akt inhibitors and discuss the potential biomarkers that may help personalized treatment of cancer with Akt inhibitors. In addition, we also discuss how Akt may confer the vulnerability of cancer cells to some kinds of anticancer agents.

## Introduction

**Fig. 1:**
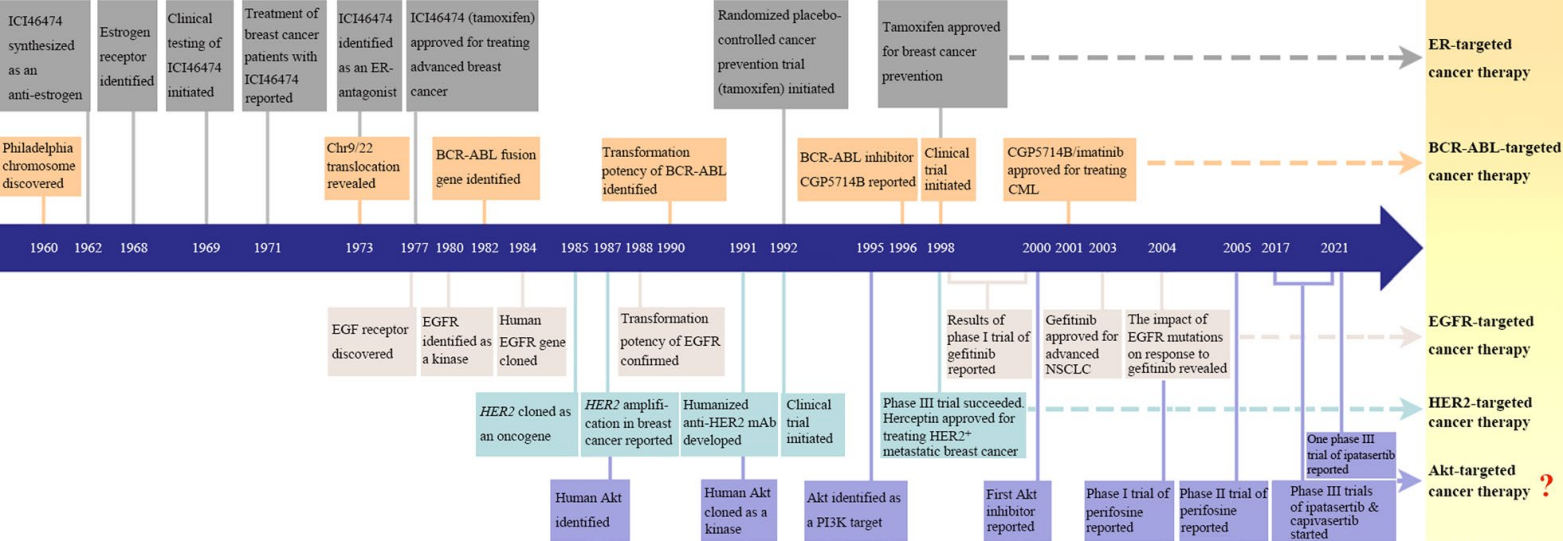
Timeline of key progresses in ER-, Bcr-Abl-, EGFR-, HER2- and Akt-targeted cancer therapy. Based on the identification of ER, Bcr-Abl, EGFR, HER2 and Akt in promoting tumorigenesis, ER-, Bcr-Abl-, EGFR- and HER2-targeted therapy have been approved for treating various types of cancer, while Akt inhibitors are still being evaluated in phase III clinical trials

Molecular targeted therapy has been widely introduced into clinical practice to treat different types of human cancer [1–5]. One of the most successful molecular targeted cancer therapeutics is the anti-estrogen therapy for patients with estrogen receptor (ER)-positive breast cancer (Fig. 1) [6–8]. However, the importance of molecular targeted therapy was not well recognized until the deregulation of protein kinases in cancer was clarified. The development of selective protein kinase inhibitors to treat cancer with aberrant activation of specific signaling pathways has been considered a promising approach since 1990s. BCR-ABL, cyclin-dependent kinases (CDKs), epidermal growth factor receptor (EGFR), epidermal growth factor receptor-2 (HER2/neu/ERBB2) and vascular endothelium growth factor receptor (VEGFR) have been identified as targets for the development of selective inhibitors. The *BCR-ABL* fusion gene, which is created through chromosomes 9 and 22 translocation (also called Philadelphia chromosome), is a key oncogene in chronic myeloid leukemia (CML) [9–11]. The Abl tyrosine kinase inhibitor imatinib (CGP 57148B/STI571) was first introduced in 1996, tested in the clinic from 1998 and approved for treating *BCR-ABL*-positive CML, Philadelphia chromosome-positive acute lymphoblastic leukemia and gastrointestinal stroma tumor [12–15].

While imatinib is a small-molecule inhibitor, monoclonal antibodies has been developed to target HER2, an oncogene that is amplified in some types of cancer [16–24]. The series of preclinical studies and clinical trials led to the regulatory approval of Herceptin/trastuzumab, a humanized monoclonal antibody targeting HER2, to treat breast cancer (Fig. 1). HER2 amplification or overexpression is the biomarker to allow precision treatment with Herceptin. *EGFR/ERBB1*, the first EGFR family member, was cloned just one year before *HER2* and then identified as an oncogene [25–28]. EGFR inhibitors such as gefitinib and erlotinib have been developed to treat cancer [29–31]. Based on the encouraging results from the phase II trials in advanced non-small-cell lung cancer (NSCLC) patients [32, 33], gefitinib received accelerate approval by the US Food and Drug Administration (FDA) to treat advanced NSCLC in 2003, followed by the approval of erlotinib (Tarceva) in 2004 [34]. However, the later disclosed phase III trials of gefitinib were disappointing [35], which led to the restriction of gefitinib use by the U.S. FDA [36]. Nevertheless, subsequent studies demonstrate that patients with sensitizing mutations of *EGFR* gene are more responsive to gefitinib or erlotinib compared with patients without such mutations [37–39], which leads to the administration of EGFR inhibitors for precision cancer therapy. In contrast, some mutations (e.g., T790M) in EGFR lead to resistance of NSCLC to gefitinib [40]. So far, the EGFR tyrosine kinase inhibitors gefitinib, erlotinib, afatinib, osimertinib and dacomitinib have been approved as first-line treatment for metastatic EGFR mutant patients with NSCLC [41]. The lessons from development of EGFR inhibitors highlight the importance of predictive biomarkers in guiding precision cancer therapy. Over the last two decades, a number of tyrosine kinase inhibitors with different targets have been developed and administered in precision cancer therapy [29, 42].

The phosphatidylinositol 3'-kinase (PI3K)/Akt pathway is one of the critical signaling outputs of Bcr-Abl, EGFR and HER2. Human homologs of *v-Akt* oncogene were preliminarily identified in 1987 and fully cloned, sequenced and characterized as a protein kinase in 1991 [43–46]. Subsequently, Akt was identified as a target of PI3K in 1995 [47]. The third member of Akt family, Akt3, was cloned firstly from a rat brain cDNA library and then cloned from human tissues [48, 49]. Of note, the three isoforms of Akt have different and even opposing functions in tumorigenesis [50, 51]. In general, Akt signaling is aberrantly activated in many cancers. Amplification of *AKT1*, *AKT2* and *AKT3* has been reported in gastric, ovarian, pancreatic and breast cancers, glioma and melanoma [44, 52–58]. In addition, overexpression of individual Akt isoforms in the absence of gene amplification has been detected in breast cancer, colorectal cancer, hepatocellular carcinoma and melanoma [59–62]. Somatic activating mutations in Akt1 and Akt3 also contribute to the hyperactivation of Akt in some types of tumors [63–67]. Akt activation promotes tumor progression and resistance to many chemotherapeutic agents [68–70]. Therefore, Akt is considered as a rational target for cancer treatment. The first Akt inhibitor ML-9 (1-(5-chloronaphthalene-1-sulphonyl)-1H-hexahydro-1,4-diazepine) were reported in 2000 [71]. However, this inhibitor also inhibits myosin light-chain kinase and stromal interaction molecule 1 [72, 73]. On the basis of the protein kinase A (PKA) inhibitor H-89, a new compound NL-71-101 was developed to inhibit Akt 2.4-fold better than PKA in 2002 [74]. The synthesis from l-quebrachitol of a series of 3-deoxygenated ether lipid-type phosphatidylinositol analogues as Akt inhibitors was reported in 2003 [75]. One year later, the results of phase I clinical trial for one of the Akt inhibitors, perifosine, was reported [76]. Currently, some Akt inhibitors, such as capivasertib (AZD5363) and ipatasertib, are being evaluated in phase III clinical trials. Herein, we review our current knowledge about Akt signaling in cancer and update recent progress in the clinical trials of Akt inhibitors. We also discuss how Akt may confer vulnerability to some kinds of anticancer agents and how gene mutations may render cancer cells vulnerable to Akt inhibition. These may help realize the goal of personalized medicine and shed new light on how to treat cancer precisely by exploiting Akt as a biomarker or molecular target [77].

## Mechanisms of Akt activation and inactivation

**Fig. 2:**
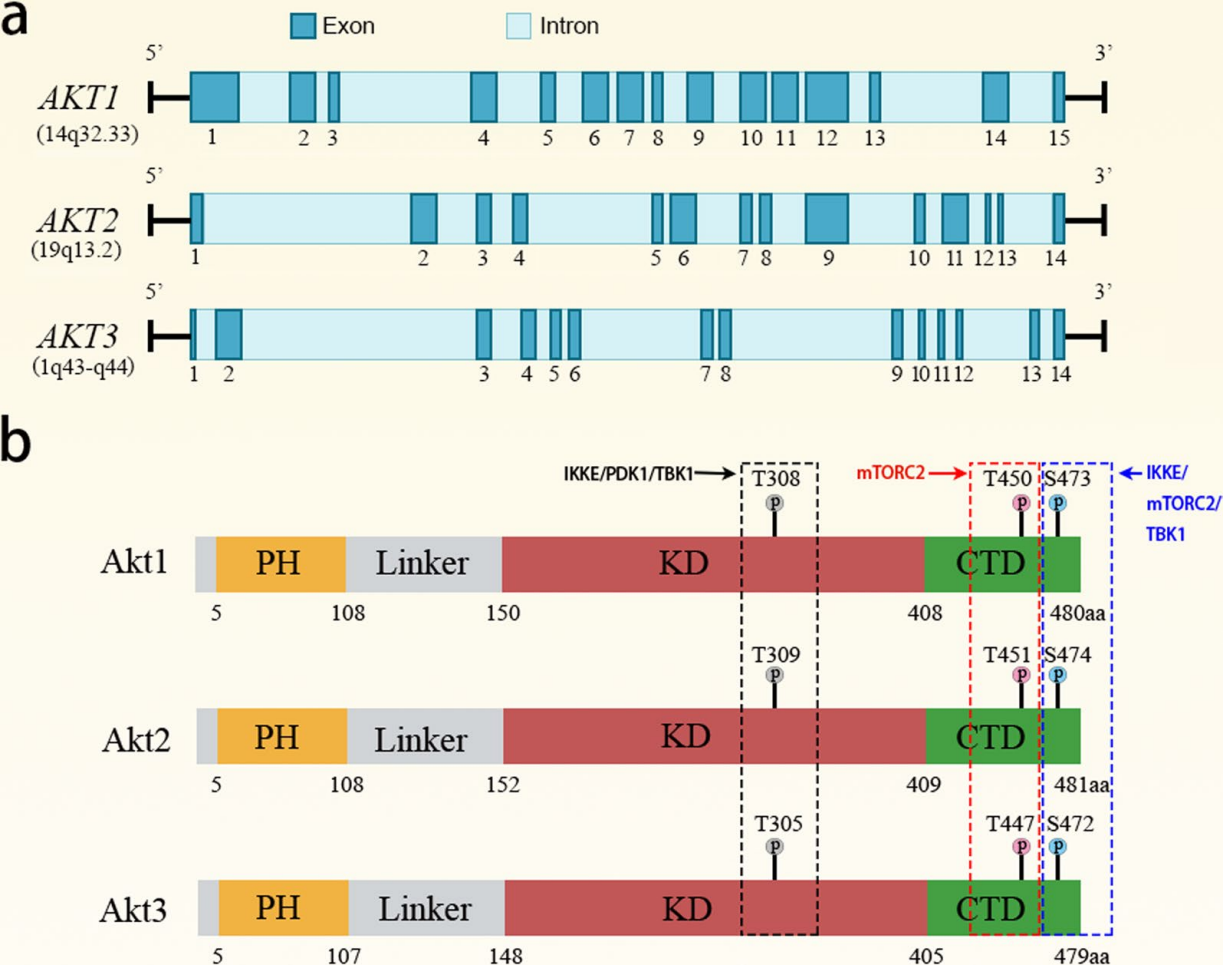
An overview of *AKT1/2/3* genes and proteins. **a** Human *AKT1* gene has 15 exons, while *AKT2* and *AKT3* gene have 14 exons. **b** Akt protein has 3 domains, the plecstrin homology (PH) domain, kinase domain (KD) and C-terminal domain (CTD). The three conserved phosphorylation sites in Akt1, Akt2 and Akt3, and the kinases that phosphorylate these residues are shown

The three members of Akt family are encoded by 3 genes (*AKT1*, *AKT2* and *AKT3*)*,* which are located at 14q32.33, 19q13.2 and 1q43-q44, respectively (Fig. 2a). Akt consists of three domains, including an N-terminal pleckstrin homology (PH) domain, a central kinase domain containing an activation loop, and an AGC kinase C-terminal domain (Fig. 2b). Structurally, the PH domain interacts with the kinase domain and elicits intramolecular autoinhibition of Akt kinase activation [78]. The PH domain is a docking site for phosphatidylinositol 3,4,5-trisphosphate (PIP3) or phosphatidylinositol 3,4-bisphosphate (PIP2). Whereas PIP3 engagement of PH domain reportedly promotes Akt activation [79], other studies indicate that PIP3 binding to the Akt1 PH domain per se does not induce Akt1 activation [80, 81]. Rather, PIP3 engagement of the PH domain may facilitate the membrane translocation of Akt and subsequent phosphorylation by PDK1, mTORC2 and other kinases [80]. Of note, a basic patch in the linker between PH and kinase domains can interact with phosphorylated C-terminal residue S473, thereby promoting Akt1 activation [81]. While the basic residue arginine in the basic patch is conserved in Akt1, Akt2 and Akt3, it remains to know whether this residue is also critical for activating Akt2 and Akt3.

Various types of stimuli including growth factors, cytokines, hormones and stresses can activate Akt. The activity of Akt is tightly regulated by post-translational modifications such as phosphorylation, ubiquitination, acetylation and palmitoylation. Dynamic activation of Akt is dictated by post-translational modifications and removal of these modifications. Although the plasma membrane distribution is critical for Akt activation, Akt can be activated at other subcellular compartments such as the endosome, lysosome, endoplasmic reticulum and nucleus. A detailed discussion of the activation of Akt at these different compartments is beyond the scope of this review. A recent review explicitly introduced the compartment-specific activation of Akt [82].

### Activation of Akt by posttranslational modification

In general, receptor- or non-receptor tyrosine kinases, PI3K, the 3-phosphoinositide-dependent protein kinase 1 (PDK1) and mTOR complex 2 (mTORC2) can activate Akt by promoting the phosphorylation of Akt1, Akt2 and Akt3 at residues T308/S473, T309/S474 and T305/S472, respectively [83]. As a catalytic product of PI3K, PIP3 is a critical activator of PDK1 and Akt. The binding of PIP3 or PI(3,4)P2 to the PH domains of Akt and PDK1 triggers membrane localization of Akt and PDK1 [84]. PDK1 directly phosphorylates Akt1 at T308, while mTORC2 not only directly phosphorylates Akt1 at S473, but also phosphorylates and activates insulin receptor (InsR)/insulin-like growth factor receptor (IGF-IR) through the tyrosine kinase activity of mTOR, thereby indirectly promoting Akt activation [83, 85]. Except for PDK1 and mTORC2, IkappaB kinase epsilon (IKKE) and TANK-binding kinase 1 (TBK1) may directly phosphorylate Akt at S473 and T308, which is dependent on PI3K but independent of PDK1 and mTORC2 [86–88]. Meanwhile, phosphorylation of C-terminal S477/T479 residues by CDK2/cyclin A2 or mTORC2, together with phosphorylation of S473, synergistically promotes the phosphorylation of Akt1 by PDK1 [81, 89]. Mechanistically, C-terminal phosphorylations can abrogate the intramolecular autoinhibition of Akt by the PH domain [81].

In addition, Mer tyrosine kinase (MERTK) can phosphorylate tyrosine 26 (Y26) of Akt1 and facilitate the activation of Akt by PI3K pathway [90]. Of note, it was reported that heat shock could induce Akt1 activation without plasma translocation, phosphorylation at S473 and T308 and active PI3K [91]. However, another study indicates that Akt phosphorylation is induced by heat shock [92]. Hence, it remains to know whether heat shock induces Akt1 phosphorylation on residues other than S473 and T308, and if kinases other than PI3K, PDK1 and mTORC2 mediate this process. Moreover, one study indicates that heat shock-induced activation of monomeric Akt is dependent on PI3K, whereas activation of oligomeric Akt upon heat shock is independent of PI3K [93].

Except for heat shock, oxidative stress may induce Akt activation through the non-receptor tyrosine kinase Src [93]. In addition, cyclic AMP (cAMP), an important second messenger, may promote Akt activation via PKA and exchange protein directly activated by cAMP [94–97]. Together, these studies indicate that the PDK1- and mTORC2-independent mechanisms of Akt activation are complex. Elucidation of such mechanisms may be important for exploring how cancer cells become resistant to PI3K/mTOR inhibition. Moreover, the Akt C-terminal modulatory protein was initially identified as an Akt inhibitor [98]. However, later studies indicate that it is overexpressed in breast cancer, head and neck cancer and positively regulates Akt phosphorylation [99, 100]. The reasons for discrepancy among these studies are unclear.

Besides Akt phosphorylation, K63-linked ubiquitinylation of Akt may promote Akt activation by facilitating membrane distribution. Tumor necrosis factor receptor associated factor (TRAF) 4, TRAF6 and S-phase kinase-associated protein 2 (Skp2) mediate K63-linked ubiquitination of Akt [101–103]. Furthermore, K63-linked polyubiquitination of Akt is promoted by SET domain bifurcated histone lysine methyltransferase 1-dependent Akt1 methylation at lysine 64, which facilitates lysine demethylase 4A-mediated recruitment of TRAF6 or Skp2 [104]. On the other hand, the deubiquitinating enzyme CYLD and ubiquitin specific peptidase 1 can remove K63-linked polyubiquitin chains on Akt and then inhibit Akt activation [105, 106]. Thus, the ying-yang balance of Akt activity can be maintained by Akt ubiquitination and deubiquitination.

Akt SUMOylation is another mechanism of Akt activation. The SUMO-conjugating enzyme Ubc9, SUMO-activating enzyme SAE1 and SUMO E3 ligase PIAS1 jointly promote Akt SUMOylation [107–109]. While the dependency on phosphorylation for Akt SUMOylation is controversial [110, 111], Akt SUMOylation enhances Akt activity [103]. Of note, SUMO and activation of SUMOylated AKT may be independent of PI3K or AKT distribution to the cell membrane [108]. On the other hand, activated Akt phosphorylates SUMO1 at T76 and Ubc9 at T35, leading to an increase in global SUMOylation [111].

Overall, it seems that Akt can be activated in PI3K-dependent or -independent manner. The binding of PIP3 to the PH domain, or co-operative modifications of Akt by protein kinases, ubiquitin ligases and SUMOylation enzymes can promote the membrane distribution of Akt and abrogate the intramolecular autoinhibition of Akt by the PH domain and further enhance the kinase activity of Akt.

### Mechanisms of Akt inactivation and degradation

While PDK1, mTOR, TBK1 and MERTK are Akt-phosphorylating kinases, the lipid phosphatases such as phosphatase and tensin homolog deleted on chromosome 10 (PTEN) and Src homology 2 domain-containing inositol-5-phosphatase (SHIP) can inhibit the phosphorylation of Akt by PI3K pathway [112–114]. Mechanistically, PTEN and SHIP convert PIP3 into PI(4,5)P2 and PI(3,4)P2, respectively. In addition, Akt can be directly dephosphorylated by PH domain leucine-rich repeat protein phosphatases (PHLPP) and protein phosphatase 2A (PP2A). Both PHLPP1 and PHLPP2 can dephosphorylate and inactivate Akt [115]. Meanwhile, the interaction between PHLPP and Akt is regulated by other proteins. ERBB receptor feedback inhibitor 1 interferes with the interaction between PHLPP and Akt thereby promoting Akt activation [116]. In contrast, sirtuin 7 promotes the interaction among FKBP51, Akt and PHLPP by deacetylating FKBP51 at lysines 28 and 155, leading to Akt dephosphorylation [117]. On the other hand, PP2A forms a complex with receptor for protein kinase 1 (RACK1) to dephosphorylate Akt [118]. Aldolase B facilitates the recruiting of PP2A to phosphorylated Akt and then promotes Akt dephosphorylation independent of the enzyme activity of adolase B [119]. Furthermore, the stability of PP2A subunits is enhanced by WNK lysine-deficient protein kinase 1, which interacts with protein phosphatase 2 scaffold subunit alpha [120]. However, Akt may reciprocally inactivate PP2A via microtubule-associated serine/threonine kinase-like (MASTL) [121]. Moreover, inhibitor 1 of PP2A (I1PP2A/ANP32A), inhibitor 2 of PP2A (I2PP2A/SET) and cellular inhibitor of PP2A (CIP2A) are oncoproteins that directly bind to PP2A and inhibit its activity [122, 123]. The micropeptide encoded by a so-called long non-coding RNA LIN00665 binds to CIP2A and inhibits its activity [124]. Hence, the activity of Akt may be determined by the balance among these regulatory elements. Except for inactivation of Akt by dephosphorylation, small ubiquitin-like modifier-specific protease 1 (SENP1), SENP2 and SENP3 can de-SUMOylate and inactivate Akt [125, 126].

While K63-linked ubiquitination of Akt positively regulates Akt activity, ubiquitinated Akt may be subject to proteasomal or lysosomal degradation. K48-linked polyubiquitination of Akt1 by zinc and ring finger 1, tetratricopeptide repeat domain 3, tripartite motif containing 13 and mitochondrial E3 ubiquitin protein ligase 1 leads to its degradation by the proteasome [127–130]. After Akt1 is sequentially ubiquitinated at lysines 284 and 214, the arginylated HSPA5 (GRP78/BIP) can promote lysosomal degradation of K48-linked ubiquitinated form of Akt [131]. In contrast, the deubiquitinase USP7 inhibits Akt ubiquitination at lysine 284/214 and lysosomal degradation [130]. Meanwhile, phosphorylation of Akt1 at T92/450 residues is essential for binding to the peptidyl-prolyl isomerase Pin1, which prevents the proteasomal degradation of Akt1 [131]. In addition, the deubiquitinase BRCA1-associated protein 1 (BAP1) reportedly stabilizes phosphorylated Akt by antagonizing its ubiquitination [132]. C-terminally truncated form of mutant ASCL1 (additional sex combs-like protein 1) interacts with BAP1 to deubiquitinate and stabilize phosphorylated Akt [132]. However, another study suggests that BAP1 may deubiquitinate and stabilize PTEN, leading to Akt inhibition [133]. Hence, the effect of BAP1 on Akt activity is inconclusive. Except for polyubiquitination, acetylation of Akt1 at lysine 14/20 by histone acetyltransferase P300 and lysine acetyltransferase 2B inhibits Akt activation [134]. Deacetylation of Akt1 by sirtunin 1 restores the activity of Akt1 [134]. Finally, caspase-mediated Akt cleavage is another mechanism of Akt inhibition [135]. Inhibition of Akt by caspase may promote cell apoptosis.

## The targets and functions of Akt

**Fig. 3:**
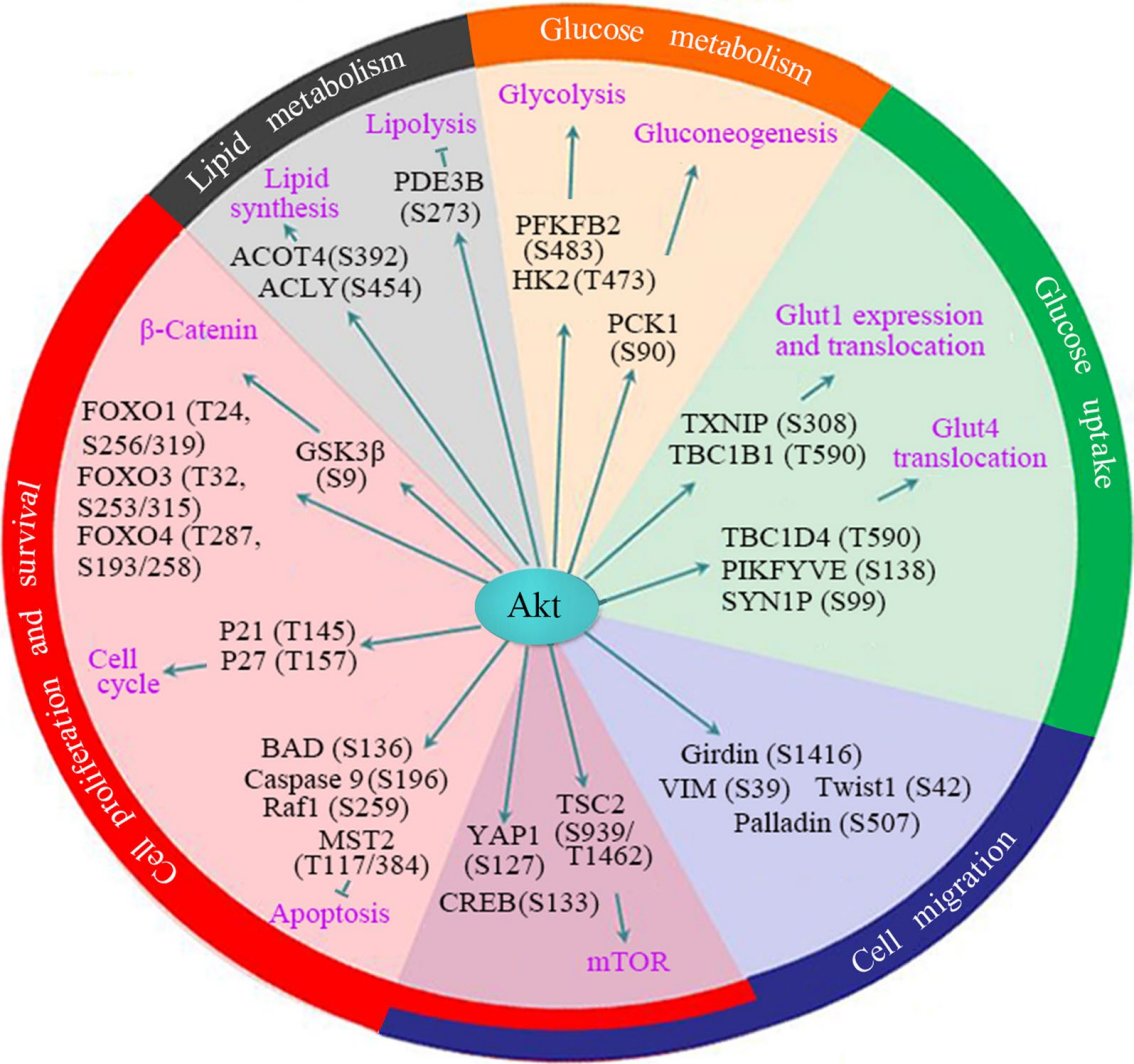
The targets and functions of Akt in tumorigenesis. Akt can directly phosphorylate its substrates in many signaling pathways and then regulate cell proliferation, survival, migration, glucose and lipid metabolism. The phosphorylation sites in the substrates of Akt are shown

Akt can directly phosphorylate many proteins that are involved in diverse cellular processes, including cell proliferation, survival, migration and metabolism (Fig. 3). The list of Akt substrates has been expanding. For most of the substrates, no Akt isoform specificity has been demonstrated. Although Akt isoforms have overlapping roles in cell signaling, previous studies have suggested that the three Akt isoforms may have some different functions, which may be due in part to isoform specific tissue distribution, subcellular localization and interaction with scaffold proteins that determine substrate selectivity [136]. While Akt1 is critical for cell survival, Akt2 is essential for glucose homeostasis [137–139]. Moreover, Akt isoforms have specific roles in cancer cells signaling. Opposing roles of Akt1 and Akt2 in cell cycle progression, migration and invasion have been detected in various types of human cancer [140, 141].

Many substrates are activated upon phosphorylation by Akt, whereas some substrates are inhibited by Akt-catalyzed phosphorylation. A number of Akt substrates contain a minimal consensus motif P/R-X-R-X-X-S/T-F/L, in which X denotes any amino acid [142]. Recently, a chemical phosphoproteomics study identifies 276 phosphorylation sites in 185 proteins that can be inhibited by 5 Akt inhibitors [143], suggesting that there are a number of direct or indirect targets of Akt in cells. The full landscape of Akt-regulated networks remains to be explored.

### Akt regulation of cell proliferation and survival

Akt can phosphorylate GSK3α and GSK3β at residues S21 and S9, respectively, thereby inactivating GSK3 and suppressing β-catenin degradation [144–146]. Similarly, Akt is able to phosphorylate and inactivate forkhead box protein O (FOXO) proteins. Akt phosphorylates FOXO1 at residues T24 and S256/319, FOXO3 at T32 and S253/315, and FOXO4 at T287 and S193/258 [147]. Phosphorylation of FOXO1/3/4 by Akt may inhibit their tanscriptional activity and/or promote their degradation [147, 148]. While both GSK3 and FOXO are inhibited by Akt, GSK3 is able to promote FOXO activation [149]. Other proteins that are phosphorylated and inhibited by Akt include p21^waf1^, p27^kip1^, BAD and caspase 9 [149–154]. In addition, Akt can phosphorylate YAP1 and sequester it in the cytoplasm, thereby disabling its induction of apoptosis [155]. Moreover, phosphorylation of the pro-apoptotic kinase MST2 by Akt may inactivate MST2 and inhibit apoptosis [156]. Hence, the inactivation of GSK3, FOXO, p21^waf1^, p27^kip1^, BAD, YAP1, MST2 and caspase 9 by Akt may promote cell proliferation and survival. While Akt reportedly phosphorylates and inactivates Raf1 [157], other studies suggest that Akt activates Raf1 to inhibit apoptosis [158]. Therefore, the regulation of Raf1 by Akt and its effect on apoptosis remains controversial.

The mechanistic target of rapamycin (mTOR) pathway is another Akt target that is involved in cell proliferation and survival [83]. Mechanistically, Akt directly phosphorylates the tuberous sclerosis 2 (TSC2) at S939/T1462 and impairs its stability, leading to the release of its inhibitory effect on mTOR [159]. In turn, activated mTOR transduces the growth factors signaling to downstream targets such as p70 ribosomal S6 kinase (S6K1) and eukaryotic translation initiation factor 4E binding protein 1, leading to increased protein translation. In addition, Akt is able to phosphorylate and activate cAMP responsive element binding protein 1, which also plays important roles in tumorigenesis [97, 160].

### Akt regulation of cell migration and cancer metastasis

Akt not only promotes tumor growth but also accelerates metastasis. Phosphorylation of actin-binding protein Girdin at S1416 by Akt leads to the accumulation of Girdin at the leading edge of migrating cells, facilitation of lamellipodia formation and promotion of cell motility [161]. Thus, phosphorylation of Girdin by Akt may not only promote cancer metastasis but also enhance VEGF-induced angiogenesis [162]. In addition, phosphorylation of Twist1 by Akt promotes cancer metastasis via upregulating transforming growth factor-β2 expression [163]. Of note, the effect of Akt on cell migration may vary among the different isoforms of Akt and among different types of human cancers. Although Akt is generally considered as oncogene, both Akt1 and Akt2 paradoxically inhibit prostate cancer cell migration by downregulating β1-integrin activity [164]. Except for cancer cell autonormous Akt1, Akt1 deficiency in endothelial cells also facilitates prostate cancer metastasis due to activation of β-catenin [165]. Moreover, Akt1 inhibits head and neck carcinoma cells and NSCLC invasion and metastasis [166, 167]. While Akt1 still inhibits breast cancer cell migration, Akt2 promotes breast cancer cell migration [50]. However, Akt1 deficiency suppressed thyroid cancer invasion and metastasis in a murine model [168]. Also, Akt1 reportedly phosphorylates vimentin at S39 and then promotes soft tissue sarcoma cell migration and invasion [169]. Other studies demonstrate that Akt1 activation promotes melanoma metastasis [170, 171]. Whereas Akt2 promotes growth factors-induced epithelial–mesenchymal transition (EMT), a process that promotes cancer metastasis [172], Akt1 negatively regulates EMT [173]. On the other hand, activated Akt1 can sequestrate β-catenin to the cell membrane, thereby reducing *ZEB1* transcription and reversing EMT [174].

The cytoskeleton is critical for cell migration and invasion. Akt1 inhibits NADPH oxidase 4 (Nox4)-derived reactive oxygen species (ROS) and then downregulates diaphanous-related formin expression and F-actin remodeling [175]. Akt1, but not Akt2, phoshorylates the S507 residue of palladin, another actin-binding protein [176]. Phosphorylation of palladin by Akt1 promotes F-actin bundling and inhibits breast cancer cell migration [176]. In contrast, Akt2 promotes palladin expression [177]. However, it is unclear how Akt2-induced palladin expression may affect cancer cell migration. As an actin-bundling protein and a scaffold, palladin has versatile effects on cell migration. It is possible that the phosphorylated and the unphosphorylated palladin have opposing effects on cell migration. In addition, Akt2 inhibits the expression of metastasis suppressor 1, a negative regulator of the actin nucleation-promoting factor cortacin [178]. Together, these studies indicate that the cytoskeleton is a key target of Akt in regulating cell migration, angiogenesis and cancer metastasis.

Similar to Akt1 inhibition, Akt3 deficiency also promotes breast cancer cells migration, invasion and metastasis due to upregulation of S100A4 and increased activation of HER2 and discoidin domain receptor tyrosine kinase 1/2 [179, 180]. In the murine breast cancer cell line PyMT, the cell–cell adhesion molecule N-cadherin downregulates Akt3 and then reduces cell motility [181]. While Akt1 promotes vascular tumor growth, Akt3 inhibits both tumor endothelial cell growth and migration [182]. However, Akt3 promotes prostate cancer metastasis by inhibiting chromosome maintenance region 1 that in turn promotes the nuclear localization of peroxisome proliferator-activated receptor gamma co-activator 1 alpha (PGC1α) and mitochondrial biogenesis [183]. These data further support the versatile roles of Akt isoforms in different types of human cancers.

### Akt regulation of cancer metabolism

Regulation of cellular metabolism is another critical function of Akt. While mTOR is an important mediator of Akt’s regulation of metabolism, there are many substrates of Akt that are involved in metabolism. Glucose metabolism is tightly regulated by the PI3K-Akt pathway. Glucose transporters (GLUT) are responsible for the uptake of glucose from extracellular milieu [184]. Insulin-regulated glucose uptake and whole-body glucose homeostasis are largely mediated by GLUT4. Akt phosphorylates TBC1D4 (AS160) and inhibits its GTPase-activating activity, thereby enabling GLUT4 trafficking to the plasma membrane [185]. In addition, phosphorylation of FYVE finger-containing phosphoinositide kinase (PIKFYVE), 250 kDa substrate of Akt (AS250) and myosin 5 by Akt promotes membrane localization of GLUT4 [186, 187]. Moreover, Akt may inhibit the expression of thioredoxin interacting protein, a negative regulator of the GLUT1 plasma membrane localization, which in turn promotes the plasma membrane distribution of GLUT1 and glucose uptake in cancer cells [188]. Of note, Akt not only promotes glucose uptake, but also enhances glucose metabolism. Phosphorylation of 6-phosphofructo-2-kinase at S483 by Akt accelerates glycolysis, a hallmark of metabolic reprogramming during tumor progression [189, 190]. Phosphorylation of the glyolytic enzyme hexokinase II at T473 enhances its protection of mitochondria and prevention of cell death [191]. In addition to direct regulation of glyolytic enzymes, Akt can promote glycolysis through other effectors such as HIF1A and mTOR. Furthermore, Akt acts downstream of the electrolytic enzymes phosphorescence isomerase and glyceraldehyde-3-phosphate dehydrogenase to mediate an antiapoptotic effect [192].

Except for regulating glucose metabolism, Akt may promote lipid synthesis by direct phosphorylation of acyl-CoA thioesterase 4 at S392 and ATP citrate lyase (ACLY) at S454 [193, 194]. Activation of ACLY promotes the production of acetyl-CoA, which can be hydrolyzed by acyl-CoA thioesterase to release free fatty acids. On the other hand, Akt may inhibit lipolysis by phosphorylating phosphodiesterase 3B [195]. Furthermore, Akt indirectly promotes lipogenic gene expression though mTOR-sterol regulatory element binding transcription factor pathway [196]. The complex roles of Akt in cancer metabolism are addressed in a recent review [197].

## Akt inhibitors for precision cancer therapy

Since Akt has critical roles in many types of human tumors, the development of Akt inhibitors has been attractive for cancer therapy. Akt has been validated as a molecular target since 1990s. Overall, many allosteric Akt inhibitors, such as MK-2206 and miransertib, and ATP-competitive inhibitors (capivasertib, ipatasertib, etc.) have been developed [176]. The action of allosteric Akt inhibitors is dependent on the PH domain of Akt to maintain Akt in the inactive conformation, while ATP-competitive inhibitors directly target the kinase domain to inhibit its activity [198, 199]. Due to the differences in target binding, different classes of Akt inhibitors may have varied potency against clinically relevant Akt mutant variants [200]. Since the pan-Akt inhibitors may have some limitations such as adverse effects, isoform-specific inhibitors of Akt have been developed as potential therapeutics [201]. Structure-based design of small molecule agents that interact with various residues in PH and CAT domains of Akt isoforms may allow isoform-specific inhibition of Akt [202]. In addition, Akt1 or Akt2 nanobodies, the antigen-binding fragment of heavy-chain-only antibodies, have been developed to target Akt1 and Akt2, respectively [203, 204]. Alternatively, knockdown of individual Akt isoform in cancer cells may be achieved by small interfering RNA. Besides genetic interference of gene expression, proteolysis targeting chimera (PROTAC) is emerging as an approach to degrading proteins of interest [205]. PROTACs against specific Akt isoform may be another choice to achieve isoform specific inhibition of Akt.

Numerous Akt inhibitors have anticancer effects in preclinical investigation [206, 207] and thus move forward with clinical trials (Table 1). However, the advances in clinical evaluation of Akt inhibitors are somewhat slow. Since 2004, the results of various stages of clinical trials of Akt inhibitors have been reported [208]. Currently, several Akt inhibitors are being tested in the phase III stage (Table 2). Many Akt inhibitors have limited anticancer activity as a monotherapy in the clinic. Hence, the ongoing clinical trials on Akt inhibitors mainly explore their potential to improve the standard cancer therapy. A recent review explicitly introduced current patents on Akt inhibitors [209]. Here, we just update some recent advances in the clinical trials.

**Table 1:** Phase I/II clinical trials of some Akt inhibitors

Trial ID	Official title	Akt inhibitor	Combination	Cancer type	Phase	Efficacy	Grade ≧ 3 adverse effects	Ref
NCT01042379	ISPY-2	MK-2206	Neoadjuvant chemotherapy	Breast cancer	II	Estimated pCR: 61.8% with MK-2206 versus 35% with control for hormone receptor-negative/HER2-positive breast cancer	Rash: 22.6% (MK-2206) versus 0 (control); Hyperglycemia: 4.3% (MK-2206) versus 0 (control)	[181]
NCT02162719	LOTUS	Ipatasertib	Paclitaxel	Metastatic TNBC	II	Median PFS: 6.2 m with ipatasertib versus 4.9 m with placebo; Median PFS in the PIK3CA/AKT/PTEN-altered population: 9.0 m with ipatasertib versus 4.9 m with placebo; Median PFS in the PIK3CA/AKT/PTEN-non-altered population: 5.3 m with ipatasertib versus 3.7 m with placebo; Median OS: 23.1 m with ipatasertib versus 16.2 m with placebo in the PIK3CA/AKT/PTEN-altered population	Rash: 2% (ipatasertib) versus 0 (placebo); Neutropenia: 10% (ipatasertib) versus 2% (placebo)	[183]
NCT02301988	FAIRLANE	Ipatasertib	Paclitaxel	Stage I-IIIa TNBC	II	pCR: 17% with ipatasertib versus 13% with placebo in the overall population; 16% with ipatasertib versus 13% (placebo) in the PTEN-low population; 18% with ipatasertib versus 12% (placebo) in the PIK3CA/AKT/PTEN-altered population; rCR: 39% with ipatasertib versus 9% (placebo) in the PIK3CA/AKT/PTEN-altered population	Diarrhea: 17% versus 1%Hyperglycemia: 0 versus 0; Rash: 1% versus 1%	[194]
NCT01485861	N.A	Ipatasertib	Abiraterone	Metastatic castration- resistant prostate cancer	II	rPFS: 8.31 m with ipatasertib (200 mg) versus 6.37 m with placebo in molecularly unstratified population; Median OS: 21.5 m with ipatasertib (200 mg) versus 15.64 m with placebo; rPFS:11.1 m with ipatasertib (200 mg) versus 4.6 m with placebo in PTEN-loss population; 4.6 m with ipatasertib (200 mg) versus 5.6 m with placebo in PTEN-non-loss population	Diarrhea: 14.3% (400 mg ipatasetib), 3.4% (200 mg ipatasertib) versus 1.2% (placebo); Asthenia: 7.2% (400 mg ipatasetib), 1.1% (200 mg ipatasertib) versus 1.2% (placebo)	[195]
NCT01625286	BEECH	Capivasertib	Paclitaxel	ER ^+^/HER^−^ metastatic breast cancer	II	PFS: 10.9 m with capivasertib versus 8.4 m with placebo in the overall population; PFS: 10.9 m with capivasertib versus 10.8 m with placebo in the PIK3CA-mutated population	Diarrhea: 22% with capivasertib versus 2% with placebo; Hyperglycemia:13% with capivasertib versus 0% with placebo; Maculopapular rash: 9% with capivasertib versus 0% with placebo	[186]
NCT01992952	FAKTION	Capivasertib	Fulvestrant	Aromatase inhibitor-resistant, advanced ER^+^/HER2^−^ breast cancer	II	PFS: 10.3 m with capivasertib plus fulvestrant versus 4.8 m with placebo plus fulvestrant in the overall population; Objective response: 29% in capivasertib group versus 8% in placebo group; CBR: 55% in capivasertib group versus 41% in placebo group; PFS: 10.3 m with capivasertib plus fulvestrant versus 4.8 m with placebo plus fulvestrant in the PI3K/PTEN pathway-nonaltered population; PFS: 9.5 m with capivasertib plus fulvestrant versus 5.2 m with placebo plus fulvestrant in the PI3K/PTEN pathway-altered population; OS: 30.5 m with capivasertib plus fulvestrant versus 18.7 m with placebo plus fulvestrant in the PI3K/PTEN pathway-altered population; 23.7 m with capivasertib plus fulvestrant versus 20.3 m with placebo plus fulvestrant in the PI3K/PTEN pathway-nonaltered population	Hypertension: 32% in capivasertib group versus 24% in placebo group Diarrhea: 14% in capivasertib group versus 4% in placebo group; Rash: 20% in capivasertib group versus 0 in placebo group; Infection: 6% in capivasertib group versus 3% in placebo group	[187]
NCT02423603	PAKT	Capivasertib	Paclitaxel	Untreated metastatic TNBC	II	PFS: 5.9 m with capivasertib plus paclitaxel versus 4.2 m with placebo plus paclitaxel in the overall population; PFS: 9.3 m with capivasertib plus paclitaxel versus 3.7 m with placebo plus paclitaxel in the PIK3CA/AKT1/PTEN-altered population; PFS: 5.3 m with capivasertib plus paclitaxel versus 4.4 m with placebo plus paclitaxel in the PIK3CA/AKT1/PTEN-nonaltered population; OS: 19.1 m with capivasertib plus paclitaxel versus 12.6 m with placebo plus paclitaxel in the overall population; Median duration of response: 13.3 m with capivasertib plus paclitaxel versus 3.5 m with placebo plus paclitaxel in the PIK3CA/AKT1/PTEN-altered population	Diarrhea: 13% (capivasertib) versus 1% (placebo); Rash: 4% (capivasertib) versus 0% (placebo) Infection: 4% (capivasertib) versus 0% (placebo) Hyperglycemia: 1.5% with capivasertib versus 0% with placebo	[191]
NCT01226316	N.A	Capivasertib	None	Akt1 E17K mutant tumors	II	Median PFS: 5.5 m in ER^+^ breast cancer patients; 6.6 m in gyneologic cancer papatients; 4.2 m in other cancer patients	Hyperglycemia: 24%; diarrhea: 17%; rash: 15.5% Discontinuation rate: 12%	[199]
NCT01226316	N.A	Capivasertib	Fulvestrant	Akt1 E17K mutant/ER^+^ metastatic breast cancer	I	Combination therapy: ORR, 36%; CBR24, 50% in fulvestrant-pretreated patients; ORR, 20%; CBR24, 47% in fulvestrant-naïve patients. Capivasertib monotherapy: ORR, 20%	Hyperglycemia: 30%; Rash: 20%	[201]
NCT00700882	NCI-MATCH (EAY131-Y)	Capivasertib	None	Akt1 E17K mutated metastatic tumor	II	ORR: 28.6%; CR: 1/35; PR: 9/35; Median duration of response: 4.4 m; SD: 46%; PD: 2/35; Median PFS: 5.5 m; Overall 6-month PFS: 50%; Median OS: 14.5 m	Hyperglycemia: 26%; Maculopapular rash: 11%	[200]
NCT02299648	VICTORY	Capivasertib	Paclitaxel	PIK3CA-mutated/amplified gastric cancer	N.A	ORR: 33.3% (8/24); 50% in PIK3CA E542K mutant population, 18.8% in the non-E542K cohort		[202]

CBR: clinical benefit rate; CR: complete response; ORR: objective response rate; OS: overall survival; pCR: pathologic complete response; PFS: progression-free survival; rPFS: radiographic progression-free survival; SD: stable disease; N.A.: not available

**Table 2:** Active or completed phase III clinical trials of Akt inhibitors

Trial ID	Official title	Akt inhibitor	Combination	Cancer type	Phase	Molecular markers	Estimated enrollment
NCT03997123	CAPItello-290	Capivasertib	Paclitaxel	TNBC	III	ER^−^/PR^−^/HER2^−^	924
NCT04862683	CAPItello-292	Capivasertib	Fulvestrant Palbociclib	Locally advanced or metastatic breast cancer	III	HR^+^/HER2^−^	628
NCT04305496	CAPItello-291	Capivasertib	Fulvestrant	Locally advanced or metastatic breast cancer	III	HR^+^/HER2^−^	834
NCT04493853	CAPItello-281	Capivasertib	Abiraterone	Hormone sensitive prostate cancer	III	AR	1000
NCT03072238^a^	IPATential150	Ipatasertib	Abiraterone Prednisolone	Metastatic prostate cancer	III	PTEN	1101
NCT03337724	IPATunity130	Ipatasertib	Paclitaxel	Locally advanced or metastatic breast cancer	III	PIK3CA/AKT1/PTEN ER^−^/PR^−^/HER2^−^HR^+/^HER2^−^	580
NCT04060862	IPATunity150	Ipatasertib	Fulvestrant Palbociclib	Locally advanced or metastatic breast cancer	III	HR^+^’HER2^−^	N.A
NCT04650581	FINER	Ipatasertib	Fulvestrant	Advanced breast cancer following progression on first-line CDK4/6 inhibitor and aromatase inhibitor	III	ER^+^/HER2^−^	250
NCT04177108	N.A	Ipatasertib	Atezolizumab Paclitaxel	Locally advanced or metastatic breast cancer	III	ER^−^/PR^−^/HER2^−^	242^b^

N.A. not available

^a^This trial has been finished.

^b^The actual enrollment number as updated in January 2021

The phase I clinical trials demonstrate that the pan-Akt inhibitors are generally tolerable in cancer patients either as a monotherapy or in combination with chemotherapy, while these drugs have some side effects including diarrhea, hypertension, rash, hyperglycemia and fatigue [210, 211]. Combination of MK-2206 with the aromatase inhibitor anasterozole appears to be unable to enhance the efficacy of anasterozole in patients with *PIK3CA*-mutant ER^+^/Her2^−^ breast cancer [212]. However, results from the I-SPY 2 trial demonstrate that the pathologic complete response rate in patients with HER2^+^ breast cancer treated with MK-2206 and standard neoadjuvant is improved when compared to neoadjuvant therapy alone, predicting a high probability of success in phase III trial [213]. In patients with locally advanced or metastatic gastric cancer and gastric-esophageal junction cancer, combination of the ATP-competitive pan-Akt inhibitor ipatasertib with leucovorin, 5-FU and oxalipatin does not improve the progression-free survival even in PTEN-low and PI3K/Akt pathway-activated subgroup [214]. For patients with metastatic triple negative breast cancer (TNBC), combination of ipatasertib with paclitaxel moderately increases the progression-free survival and overall survival when compared to treatment with placebo and paclitaxel in the LOTUS trial [215]. However, the FAIRLANE trial suggests that no significant improvement in pathologic complete response is achieved by combined treatment of stage I-IIIa TNBC with ipatasertib and paclitaxel compared to paclitaxel monotherapy, even though there is significant improvement in the MRI complete response rate [216]. In addition, combined treatment with ipatasertib and abiraterone may improve the radiographic progression-free survival in patients with metastatic castration-resistant prostate cancer when compared to abiraterone monotherapy, especially in PTEN-deficient tumors [217]. However, there is no association between tumor PTEN loss and RECIST overall response rate, circulating tumor cell reduction and prostate-specific antigen response [217]. It warrants further trials to evaluate this regimen in castration-resistant prostate cancer patients.

While the BEECH trial shows that combination of the ATP-competitive pan-Akt inhibitor capivasertib with paclitaxel, compared to paclitaxel monotherapy, has no significant improvement in the median progression-free survival of patients with advanced or metastatic ER^+^/HER2^−^*/*PIK3CA-mutant breast cancer [218], the FAKTION trial demonstrates that combined treatment of aromatase inhibitor-resistant, advanced or metastatic ER^+^/HER2^−^ breast cancer with capivasertib and fulvestrant significantly increased the progression-free survival [219]. Further evaluation of the data from this phase II trial by restricted mean survival time analysis indicates that this regimen may also improve the overall survival compared to fulvestrant monotherapy [220, 221]. In addition, this regimen seems to be effective in both PI3K pathway-altered and PI3K pathway-unaltered groups, while it is unclear whether the changes in this pathway can be accurately detected [220, 221]. Another cell-free circulating tumor DNA (ctDNA) testing-guided clinical trial, which also includes the combined treatment of breast cancer with capivasertib and fulvestrant, indicates that ctDNA testing rapidly and accurately detects gene mutations [222]. Moreover, combined treatment of TNBC with capivasertib and paclitaxel significantly improves the progression-free survival and overall survival compared to treatment with placebo and paclitaxel [223]. Biomarker analysis shows that treatment of ER^+^ breast cancer with capivasertib (360 mg or 480 mg twice a day) for 4.5 days effectively inhibits the phosphorylation of Akt targets including GSK3 and PRAS40 [224].

Genomic profiling-guided precision cancer therapy is an attractive strategy. *AKT1* mutation occurs in many cancers at a low prevalence (1% across diverse solid tumors) [225]. The frequency of *AKT1* mutations may vary among different populations. The oncogenic *AKT1*^E17K^ mutation is detected in only 1.4% of breast cancer patients who are primarily Caucasian and Hispanic [226]. While *AKT1*^E17K^ is absent in the breast cancer subset of the Cancer Genome Atlas (TCGA) datasets, it is detected in appropriately 6% of a population of Chinese breast cancer patients and a cohort of endocrine-resistant and ER^+^ metastatic breast cancer [227, 228]. In addition, 12% of carcinosarcomas have *AKT2* alterations [229]. In the TCGA datasets, *AKT3* amplification is detected in approximately 10% of breast cancer patients [228, 230]. *AKT1*^E17K^- mutant tumors are resistant to allosteric Akt inhibitors, but sensitive to ATP-competitive inhibitors. A basket study of capivasertib in heavily pretreated patients with *AKT1*^E17K^-mutant tumors demonstrates that the median progression-free survival ranges from 4.2 to 6.6 months in patients with ER^+^ breast, gynecologic and other solid tumors [231]. The most common grade ≥ 3 adverse events were hyperglycemia (24%), diarrhea (17%) and rash (15.5%) [230]. In the subprotocol EAY131-Y of US National Cancer Institute Molecular Analysis for Therapy Choice (NCI-MATCH) trial, the effects of capivasertib in heavily pretreated patients with metastatic tumors harboring *AKT1*^E17K^ mutation were evaluated [232]. Among 35 evaluable and analyzable patients in this trial, the objective response rate was 28.6% including one complete response, and approximately half of the patients had stable disease, while 2 (6%) had progressive disease [232]. Another phase I clinical trial of capivasertib monotherapy or combined treatment with fulvestrant in 63 heavily pre-treated patients with *AKT1*^E17K^/ER^+^ metastatic breast cancer demonstrated that the objective response rate was 20% in 20 patients receiving monotherapy, 36% in fulvestrant-pretreated patients with combined treatment with capivasertib and fulvestrant, and 20% in fulvestrant-naïve patients with combined treatment [233]. In addition, a more than 50% decrease in *AKT1*^E17K^ at cycle 2 day 1 appears to be associated with improved progression-free survival [233]. Surprisingly, the adverse effects are less severe in patients treated with both capivasertib and fulvestrant when compared to caspivasertib monotherapy (Table 1).

In the VIKTORY Umbrella Trial, patients with metastatic gastric cancer were recruited to receive tumor genomic profiling-guided therapy [234]. While the response rate is less than 15% in *PIK3CA*-unmutated patients receiving capivaserib monotherapy, the objective response rate is 33.3% (8/24) in patients with *PIK3CA*-mutated/amplified tumors receiving capivasertib/paclitaxel therapy [234]. Of note, this study indicates that *PIK3CA*^E542K^ patients may have a more profound decrease in tumor burden and increased response rate compared to patients with other point mutations in *PIK3CA* (Table 1)*.* However, these data should be interpreted with caution, due to the small sample size in individual groups. Randomized clinical trials with larger sample size are warranted to validate these data.

Based on the results from some phase II clinical trials, several phase III clinical trials of capivasertib and ipatasertib have been initiated (Table 2). The CAPItello series of trials aim to evaluate the efficacy of combined treatment with capivasertib and paclitaxel, palbociclib, fulvestrant or abiraterone in breast and prostate cancer patients (clinicaltrials.gov; NCT03997123/NCT04862683/NCT04305496/NCT04493853). In parallel, the IPATunity 130/150 and FINER trials aim to evaluate the efficacy of combined treatment with ipatasertib and paclitaxel, palbociclib, fulvestrant or abiraterone/prednisone (clinicaltrials.gov; NCT03072238/NCT03337724/NCT04660862/NCT04650581/NCT04177108). Recently, the results of IPATential150, a randomized and double-blind trial, demonstrate that combination of ipatasertib with abiraterone and prednisolone may significantly improve radiographic progression-free survival compared with placebo plus abiraterone among patients with PTEN-loss, metastatic and castration-resistant prostate cancers (18.5 m for the ipatasertib arm versus 16.5 m for the placebo arm (hazard ratio, 0.77; 95%CI 0.61–0.98) [235]. The final overall survival analysis of IPATential150 trial and the results from other randomized phase III trials may provide more powerful evidence to determine whether Akt inhibitors can improve cancer therapy.

## Biomarkers and molecular basis of response to Akt inhibitors in cancer

The sensitivity to molecular targeted therapy usually varies among individual cancer patients and at different stages of tumor progression. Elucidation of the mechanisms underpinning the sensitivity to molecular targeted agents may help identify the appropriate biomarkers to stratify patients for precision cancer therapy [236, 237]. Akt-dependent tumors may be vulnerable to therapeutic Akt inhibition. Currently, the PI3K/Akt/mTOR pathway activation in tumors has been taken into account in some clinical trials of Akt inhibitors. Either high or low levels of Akt pathway phosphoproteins may be associated with the responsiveness to Akt inhibition, depending on the cancer types. In general, it appears that the biomarker-assigned therapy has better effects than conventional therapy.

### Alterations in PI3K/Akt /mTOR pathway as biomarkers for Akt inhibition

Among the potential biomarkers for precision treatment with Akt inhibitors, alterations in PI3K/Akt pathway are generally taken into account in multiple clinical trials (Table 3). In the ISPY-2 breast cancer trial, higher levels of Akt substrate phosphorylation in HER2^+^ tumors are associated with better response to MK2206, whereas lower levels of Akt pathway phosphoproteins correlate with better response in TNBC [238]. The oncogenic *AKT1*^E17K^, the most common *AKT1* mutation, occurs infrequently in many types of cancers [226, 227, 232]. *AKT1*^E17K^-mutant is constitutively active and capable of promoting GLUT4 translocation [63]. Inhibition of Akt appears to be effective in treating tumors with *AKT1*^E17K^ and *AKT1*^Q79K^ mutants, even if these patients were heavily pre-treated [232, 233]. Notably, the imbalance of the *AKT1*^E17K^-mutant allele, most frequently present in breast and gynecological cancers, is associated with better response to capivasertib [232]. Except for Akt missense mutations, activating *AKT1/2* indels are detected in breast, prostate and clear-cell renal cancers [239]. One of the Akt indels, *AKT1* P68_C77dup, leads to high level of Akt activation and increased sensitivity to capivasertib in breast cells, indicating that this genomic alteration may be a potential biomarker of sensitivity to ATP-competitive Akt inhibitors [239].

**Table 3:** Potential biomarkers for precision treatment with Akt inhibitors

Biomarker	Cancer types	References
PTEN mutation or deficiency	Prostate cancer, breast cancer, endometrial cancer, glioblastoma, thyroid cancer, etc.	[235]
PIK3CA mutation	Breast cancer, ovarian cancer, colorectal cancer, lung cancer, endometrial carcinoma, cervical adenocarcinoma, glioma, head and neck cancer, etc.	[234]
Akt1 E17K	Breast cancer, ovarian cancer, endometrial carcinoma, meningioma, etc.	[232, 240]
Activating Akt1/2 indels	Breast cancer, prostate cancer, clear cell renal cancer, etc.	[239]
ARID1A/B mutations or deficiency	Gastric, ovarian and endometrioid carcinoma, medulloblastoma	[245–247]
SF3B1 mutation	Myelodysplastic/myeloproliferative neoplasms, melanoma, breast cancer, pancreatic cancer, prostate cancer, AML, etc.	[254, 255]
NPM1 mutation	AML	[257]
NM23-H1	Breast cancer, melanoma, etc.	[248]
CBL mutation	Myeloid neoplasmas	[249]
BTK mutation or deficiency	Follicular lymphoma	[253]
GPS2 mutation	Breast cancer, medulloblastoma	[250–252]
CDH1 mutation or deficiency	Gastric cancer, breast cancer, prostate cancer, colorectal cancer, ovarian cancer, etc.	[261, 262]
SMAD4 mutation	Colorectal cancer, pancreatic cancer	[269, 270]
GAB2 mutation or amplification	Hematological malignancies, ovarian cancer, lung cancer, neuroblastoma, melanoma, breast cancer	[264–268]

Another study demonstrates that the presence of *PIK3CA*/*AKT1* mutations and absence of alterations in *MTOR* or *TSC1* were associated with sensitivity to capivasertib monotherapy in HER2^−^ breast cancer [240]. The presence of *AKT1*^E17K^ and coincident PI3K pathway hotspot mutations may render tumors more responsive to Akt inhibition [232]. Among the many point mutations of PIK3CA, the data from VIKTORY Umbrella Trial suggest that *PIK3CA*^E542K^-mutant is more significantly correlated with the response to capivaserib compared to other *PIK3CA* mutations [234]. The reason for this differential association among the *PIK3CA* mutations remains unclear, and this scenario may need validation in larger clinical trials. In addition, copy number loss, truncation or point mutations of phosphoinositide-3-kinase regulatory subunit 1 (PIK3R1/p85α) are frequent events in some types of cancer. PIK3R1 deficiency leads to Akt activation due to an increase in the activity of P110 and a decrease in the activity of PTEN and growth factor receptor bound protein 2-associated protein 2 (GAB2) [241]. Preclinical study indicates that PIK3R1-depleted ovarian cancer cells are vulnerable to Akt inhibition [234]. While some clinical trials indicate that PTEN status is also associated with the benefit of Akt inhibition [215, 223], another study demonstrates that there is no difference in the sensitivity to capivasertib between PI3K/PTEN-altered and PI3K/PTEN non-altered cancer patients [209]. Taken into account of data from these clinical trials, the role of PTEN loss in dictating the response to Akt inhibitors remains to be clarified.

### Diverse regulators of Akt as potential biomarkers for Akt inhibition

Except for the genetic changes in the PI3K/Akt pathway, other genetic aberration that may affect Akt activation should be considered. AT-rich interactive domain 1 (ARID1) is a component of the mammalian SWI/SNF chromatin-remodeling complex that regulates gene expression. Loss-of-function mutations of ARID1A are frequently detected in various types of cancer such as gastric cancer, endometriosis-associated ovarian cancer and endometrioid adenocarcinoma [242–244]. ARID1A deficiency leads to activation of Akt and hypersensitivity to Akt inhibitor [245, 246]. Moreover, loss-of-function mutation in the *ARID1B* gene or downregulation of ARID1B expression in medulloblastoma leads to an increase in PI3K/Akt signaling due to decreased expression of negative regulators of Akt such as dual specificity phosphatase 2, thyroid transcription factor 1, leucine zipper tumor suppressor 1, DAB2 interacting protein and protein tyrosine phosphatase receptor type U [247]. It warrants further studies to determine whether ARID1A/B mutation or expression may be a potential biomarker to predict the sensitivity to Akt inhibition.

In fact, there is a long list of genetic events that affect Akt activation in cancer cells. Whether some of these aberrant changes can serve as biomarkers to predict the sensitivity to Akt inhibitors remains to be determined in clinical setting. The metastasis suppressor NM23-H1 (NME1) interacts with the class I PI3K catalytic subunit p110α and then inhibits Akt activation [248]. It remains to know whether NM23-H1 mutation or deficiency renders cancer cells sensitive to Akt inhibitors. Moreover, there are frequent mutations of casitas B-lineage lymphoma (CBL), which encodes an E3 ubiquitin ligase that regulates many tyrosine kinases, in myeloid neoplasms [249]. Phosphorylation of mutant CBL by Lyn kinase promotes its interaction with PIK3R1 and activation of PI3K/Akt signaling [249]. It warrants further study to determine if CBL mutation can predict the sensitivity to Akt inhibitors. In addition, G protein pathway suppressor 2 (GPS2) is a significantly mutated gene in breast cancer, medulloblastoma and other tumors [250–252]. GPS2 depletion in breast cancer cells results in sustained PI3K/Akt signaling and increased cell proliferation, migration and invasion, which can be inhibited by MK2206 [250]. Thus, GPS2 mutation may be a potential biomarker for the responsiveness to Akt inhibitors [250].

In follicular lymphoma, inactivating mutations in Bruton tyrosine kinase (BTK) lead to augmented Akt activation [253]. Moreover, mutations in cancer-associated splicing factor 3b subunit 1 (SF3B1) have been detected in hematological malignancies, breast cancer, prostate cancer, pancreatic ductal carcinoma, melanoma and other types of cancer [254]. SF3B1 mutants cause aberrant mRNA splicing and suppression of PPP2R5A, leading to Akt activation and the hypersensitivity to Akt inhibitors [255]. Since mutations in another splicesomal gene, SURP and G-patch domain containing 1 (*SUGP1)*, induce aberrant splicing identical or similar to that observed in mutant SF3B1 cancers, it remains to know whether *SUGP1* mutations also confer hypersensitivity to Akt inhibitors [256]. Moreover, the frameshift mutations in nucleophosmin 1 (NPM1), a genetic event in about one-third of patients with acute myeloid leukemia (AML), lead to increased Akt activation that renders hypersensitivity to Akt inhibitors [257]. Whereas the wild-type NPM1 inhibits Akt phosphorylation, the AML-associated NPM1 mutants can prevent the nuclear localization of Akt and promote Akt phosphorylation [257]. Of note, overexpression of NPM1 has been detected in solid tumors including colorectal and hepatocellular carcinoma [258]. It warrants further studies to determine whether NPM1 overexpression may affect the sensitivity to Akt inhibitors. In addition, cyclin-dependent kinase inhibitor 2A/B (CDKN2A/B) deficiency in malignant pleural mesothelioma leads to PI3K/Akt activation [259]. It remains to know whether loss of CDKN2A/B renders a priority to treatment with Akt inhibitors.

### Germline mutations as potential biomarkers for Akt-targeted cancer therapy

Individuals with Cowden syndrome, an autosomal dominant genetic disease, have a high risk of breast cancer in their lifetime. Approximately 80% of patients with Cowden syndrome have a germline inactivating mutation in *PTEN*. Kinston et al. reported an exceptional response to capivasertib in two cases with Cowden syndrome, breast cancer and germ-line *PTEN* mutations [260], indicating that Akt is a promising target for treating patients with germline *PTEN* mutations. In addition, germline mutations in cadherin 1 (*CDH1)* may lead to hereditary diffuse gastric cancer syndrome that is associated with upregulated Akt activity [261]. Preclinical study indicates that CDH1 deficiency renders gastric cancer cells vulnerable to allosteric Akt inhibitors, possibly due to the increased expression of Akt3 [262]. Of note, CDH1 mutations have been detected in many other types of cancer including breast, prostate, colorectal, ovarian and thyroid cancers [262]. It warrants clinical study to determine whether Akt inhibitors can effectively treat cancer patients with germline or somatic *CDH1* mutations.

Germline mutations may lead to the development of familial and sporadic hematological malignancies [263]. The GAB2 P621fs frameshift mutation leads to increased interaction between GAB2 and the p85 regulatory subunit of PI3K, which promotes P3K/Akt signaling [264]. Except for GAB2 mutation, GAB2 amplification or overexpression has been detected in ovarian, lung, breast cancers and melanoma [265–268]. Hence, it warrants further studies to determine if Akt inhibitors may effectively treat GAB2 mutant-associated hematological malignancies and GAB2-amplified cancers. In addition, mothers against decapentaplegic homolog 4 (SMAD4) interacts with rictor and prevents the phosphorylation of Akt by mTORC2 [269]. Hence, loss-of-function SMAD4 mutations, which are present in some types of tumors such as mucinous adenocarcinoma of the colon and pancreatic carcinoma [270], may lead to Akt activation. SMAD4-deficient colon cancer may be vulnerable to combined treatment with Akt inhibitors and irinotecan [269]. Finally, germline mutations of SAMD4 are associated with juvenile polyposis syndrome [271, 272]. It remains to know whether Akt inhibition is a potential strategy to prevent or treat cancers in individuals with germline or somatic mutations of SMAD4.

## Exploiting redox and metabolic vulnerability in Akt-driven cancer

While a significant proportion of patients with cancers harboring PI3K/Akt pathway alteration may be treated by Akt inhibitors, some of these patients may be insensitive to Akt inhibition due to primary or acquired drug resistance. In addition, Akt inhibitors may have severe adverse effects in about 30% of patients, which may lead to drug discontinuation. Although Akt activation may promote tumorigenesis, it may also make cancer cells vulnerable to some insults. Therefore, an alternative strategy to treat cancers with Akt hyperactivation may be exploiting its vulnerability.

Since Akt can promote both glycolysis and oxidative phosphorylation, the generation of ROS, a by-product of oxygen consumption and oxidative phosphorylation, may be increased in Akt-hyperactive cells [273, 274]. In addition, Akt may phosphorylate mitochondrial calcium uptake 1 (MICU1) at S124 and then inhibit mitochondrial calcium uniporter (MCU), leading to an increase in mitochondrial calcium levels and ROS [275]. NADPH is an important metabolite to maintain redox homeostasis. Activation of the NOX compromises the anti-oxidant effect of NOX and then promotes the rapid accumulation of ROS. Akt3 can activate NOX by directly phosphorylate p47^phox^, one of the NOX regulators, at S304 and S328, which results in increased levels of intracellular ROS [276]. On the other hand, previous studies demonstrate that all Akt isoforms can inhibit reactive oxygen species (ROS) scavenging, while Akt3 is the most potent isoform to induce ROS [276, 277]. In addition, the inactivation of FoxO transcriptional factors by Akt may result in decreased expression of FoxO-regulated ROS scavengers, such as SOD and sestrin 3 [274]. Hence, the intracellular levels of ROS may be increased in Akt-hyperactive cancer cells, depending on the plasticity of redox homeostasis.

ROS is a double-edge sword with both tumor-promoting effects and detrimental consequence on cell survival [278]. In fact, ROS can promote PI3K/Akt signaling through complex mechanisms including downregulation of PTEN, inhibition of protein tyrosine phosphatases and upregulation of receptor tyrosine kinases [277, 279, 280]. Akt is one of the mediators of ROS signaling [281]. On the one hand, ROS may promote cell senescence, growth arrest and cell death if the redox homeostasis is disrupted, and the cells fail to adapt to high levels of intracellular ROS [282]. Since Akt activation compromises some of the anti-oxidant elements, the growth of tumor cells expressing activated Akt may rely on alternative antioxidation system. In fact, the survival of Akt-activated cancer cells is dependent on FoxM1, which can not only downregulate ROS levels by upregulating the expression of anti-oxidant genes, but also prevent ROS-induced cell death [283]. During cancer evolution, increased ROS as a consequence of Akt activation may exert selective pressure to enable a reprogrammed antioxidant and detoxification system that allows for higher level of redox balance to favor the pro-tumorigenic effects of ROS. After acquiring a fitness advantage, Akt hyperactive cancer cells may expand over time.

Harnessing Akt-induced impairment of ROS scavenging for therapeutic development is an alternative strategy to treat tumors expressing hyperactive Akt. Further increasing intracellular ROS by β-phenylethyl isothiocyanate can kill cancer cells in which Akt activation is induced by rapamycin [274]. While Akt activates some pro-oxidant proteins and impairs selective antioxidant factors, the efficacy of ROS-increasing therapy for Akt-hyperactive tumors may depend on the plasticity of antioxidant system and the extent to which ROS levels are increased. Intracellular ROS levels may be a predictive biomarker for this therapy. The reliance of Akt-hyperactive cancer cells on antioxidant activity may be another vulnerability that can be exploited to treat cancer [284]. Further disabling key antioxidant proteins may render cancer cells more vulnerable to agents that induce the production of ROS.

## Conclusions and perspectives

As a well-studied protein kinase, Akt has been suggested to play key roles in the regulation of development, glucose homeostasis, tumor growth and metastasis. Mounting studies have provided deep insight into the mechanisms of Akt activation and inactivation, and the diverse functions of Akt in tumor progression and drug resistance. As described above, Akt can be activated in PI3K-dependent or PI3K-independent manners. The identification of molecular events associated with Akt signaling has established its role in tumorigenesis and development of new therapeutics. The successful development of allosteric or ATP-competitive Akt inhibitors and clarification of their efficacy in preclinical studies pave the way to evaluate the potential of Akt-targeted cancer therapy in the clinic. Alternatively, inhibition of Akt by some natural agents may be valuable for cancer chemoprevention and therapy [285, 286]. Since Akt activation confers resistance to anticancer agents such as the multikinase inhibitor sorafenib, the ER antagonists tamoxifen and fulvestrant [219, 287–289], combination of Akt inhibitors and other anticancer agents may improve the efficacy of cancer therapy.

In view of the latest results from multiple clinical trials, Akt inhibitors such as ipatasertib and capivasertib hold promise in treating breast and prostate cancers, especially when combined with paclitaxel, fulvestrant, abiraterone, palbociclib and atezolizumab. Most of the finished trials were conducted in heavily pretreated and advanced cancer patients with relatively small sample size. Randomized clinical trials with large sample size are warranted to confirm the efficacy of biomarker-guided treatment with Akt inhibitors in cancer patients. In addition, it remains to know the efficacy of Akt inhibitors as first-line treatment for cancer. Even though Akt inhibitors may effectively treat selective cancer patients, it needs compare the efficacy of Akt inhibitors with other agents that target different members in the related pathways, such as PI3K, mTOR and CDK.

In the era of precision cancer therapy, predictive biomarkers are critical to stratify patients who most likely benefit from a given targeted therapy. Genetic, epigenetic and proteomic changes in tumors are associated with the sensitivity to many drugs. Overall, cancer patients receiving genomically matched precision therapy have increased objective tumor response rate and better overall survival when compared to unmatched therapy [290, 291]. The same may be true for Akt inhibitors. While PTEN loss and activating Akt mutations are promising biomarkers, and most of the clinical trials for Akt inhibitors are guided by changes in PTEN, PI3K, Akt and mTOR, it warrants further studies to validate these biomarkers and identify other potential biomarkers that can help select cancer patients who may be responsive to Akt–targeted therapy.

The genetic, epigenetic and proteomic changes in tumors can be identified by local testing of tumor tissues or central testing of plasma cell-free DNA (cfDNA) by high-throughput approaches [292]. Local testing of DNA from tumor tissues can be achieved by matrix-assisted laser desorption ionization time-of-flight mass spectrometry, whole-genome array-comparative genomic hybridization or next-generation sequencing (NGS), and exon-capture NGS [291, 293]. The approach for central testing of plasma cfDNA includes BEAMing, droplet digital polymerase chain reaction and NGS [231, 294, 295]. While local testing of archival tumor tissues may be more sensitive than central testing of plasma cfDNA [231], central testing of plasma cfDNA may not only help stratify cancer patients for matched precision therapy, but also capture additional tumor heterogeneity, monitor the dynamic changes in genetic markers and evaluate the therapeutic response or predict the prognosis [296].

Of note, pan-Akt inhibitors may have severe adverse effects in some patients, which even lead to drug discontinuation. Under these circumstances, an alternative strategy may be development of isoform-specific Akt inhibitors. However, this kind of Akt inhibitors still meets many challenges. The efficacy of isoform-specific Akt inhibitors may depend on whether tumors are addictive to a given isoform of Akt. On the other hand, tumors may evolve to adapt to the selective pressure of isoform-specific Akt inhibitors by rewiring to other isoforms. Exploiting the vulnerability of Akt-hyperactive cancer cells is another strategy to treat such cancers. Except for the redox disregulation, other potentially fatal weakness of Akt-hyperactive cancer remains to be identified.

Drug resistance is a severe problem in cancer therapy. Cancer cells may resist Akt inhibition via complex mechanisms. Since Akt induces inhibitory phosphorylation of RAF1 S259 and BRAF S365 [151], inhibition of Akt may result in derepressing RAF1 and BRAF. In addition, RAF/RAS mutations may lead to ERK hyperactivation, which confers resistance to Akt inhibition. Inhibition of Akt may also lead to FOX3A-mediated upregulation of ER and IGF-IR expression [143, 297]. Furthermore, serum/glucocorticoid regulated kinase (SGK) and Akt share many substrates. SGK can sustain Akt-independent activation of signaling molecules such as mTOR [298]. Meanwhile, cyclin D1 overexpression and loss of Akt1 E17K mutation are associated with acquired resistance to capivasertib [240]. Simultaneous inhibition of Akt and other compensatory pathways may improve the efficacy.

Akt plays important roles in many physiological and pathological processes. Hence, the systemic effects of Akt inhibitors must be taken into account when they are used to treat cancer. Targeted inhibition of PI3K/Akt/mTOR pathway often disrupts glucose uptake and metabolism in some tissues, which may lead to hyperglycemia, one of the common side effects of PI3K, Akt and mTOR inhibitors [299, 300]. Hyperglycemia usually feeds back to stimulate insulin release from the pancreas. The systemic glucose-insulin feedback contributes to PI3K inhibitors resistance during cancer therapy, which can be circumvented by ketogenic diet, metformin and SGLT2 inhibitors [301]. The same may be true for Akt-targeted cancer therapy. Meanwhile, the insulin-independent glucose consumption by tumor cells may also be involved in Akt inhibitors resistance as well. On the other hand, hepatic Akt inhibition may induce liver injury, inflammation and carcinogenesis and promote lung metastasis in mice [301]. It remains to know whether long-term treatment with Akt inhibitors may induce such adverse effects in human. Tumor-targeted delivery of Akt inhibitors may relieve the systemic adverse effects and enhance the anticancer activity.

## Data Availability

Not applicable.

## Abbreviations

ACLY: ATP citrate lyase
AML: Acute myeloid leukemia
ARID1: AT-rich interactive domain 1
BAP1: BRCA1-associated protein 1
CBL: Casitas B-lineage lymphoma
CDH1: Cadherin 1
CDK: Cyclin-dependent kinases
CDKN2A/B: Cyclin-dependent kinase inhibitor 2A/B
cfDNA: Cell-free DNA
CIP2A: Cellular inhibitor of PP2A
CML: Chronic myeloid leukemia
ctDNA: Circulating tumor DNA
EGFR: Epidermal growth factor receptor
EMT: Epithelial–mesenchymal transition
ER: Estrogen receptor
FOXO: Forkhead box protein O
GAB2: Growth factor receptor bound protein 2-associated protein 2
GLUT: Glucose transporters
GPS2: G protein pathway suppressor 2
GSK3: Glycogen synthase kinase 3
HER2: Epidermal growth factor receptor-2
I1PP2A: Inhibitor 1 of PP2A
I2PP2A: Inhibitor 2 of PP2A
IGF-IR: Insulin-like growth factor receptor
IKKE: IkappaB kinase epsilon
InsR: Insulin receptor
MERTK: Mer tyrosine kinase
mTOR: Mechanistic target of rapamycin
mTORC2: mTOR complex 2
NGS: Next-generation sequencing
NSCLC: Non-small-cell lung cancer
PDK1: 3-Phosphoinositide-dependent protein kinase 1
PGC1α: Peroxisome proliferator-activated receptor gamma co-activator 1 alpha
PI3K: Phosphoinositide 3-kinase
PIK3CA: Phosphatidylinositol-4,5-bisphosphate 3-kinase catalytic subunit alpha
PIK3R1: Phosphoinositide-3-kinase regulatory subunit 1
PIP2: Phosphatidylinositol 3,4-bisphosphate
PIP3: Phosphatidylinositol 3,4,5-trisphosphate
PHLPP: PH domain leucine-rich repeat protein phosphatases
PROTAC: Proteolysis targeting chimera
PP2A: Protein phosphatase 2A
PTEN: Phosphatase and tensin homolog deleted on chromosome 10
RACK1: Receptor for protein kinase 1
ROS: Reactive oxygen species
SENP: Small ubiquitin-like modifier-specific protease
SGK: Serum/glucocorticoid regulated kinase
SHIP: Src homology 2 domain-containing inositol-5-phosphatase
SKP2: S-phase kinase-associated protein 2
SMAD4: Mothers against decapentaplegic homolog 4
SUMO: Small ubiquitin-like modifier
TBK1: TANK-binding kinase 1
TCGA: The cancer genome atlas
TNBC: Triple negative breast cancer
TRAF: Tumor necrosis factor receptor associated factor
TSC1: Tuberous sclerosis 1
TSC2: Tuberous sclerosis 2
VEGFR: Vascular endothelium growth factor receptor
